# Galcanezumab effect on “whole pain burden” and multidimensional outcomes in migraine patients with previous unsuccessful treatments: a real-world experience

**DOI:** 10.1186/s10194-022-01436-6

**Published:** 2022-06-13

**Authors:** Marcello Silvestro, Alessandro Tessitore, Ilaria Orologio, Rosa De Micco, Lorenzo Tartaglione, Francesca Trojsi, Gioacchino Tedeschi, Antonio Russo

**Affiliations:** Headache Center, Department of Advanced Medical and Surgical Sciences (DAMSS), University of Campania “Luigi Vanvitelli”, Piazza Miraglia 2 - I-80138, Naples, Italy

**Keywords:** Galcanezumab, Migraine, Monoclonal antibodies, Real-world, CGRP

## Abstract

**Background:**

Clinical trials have demonstrated galcanezumab as safe and effective in migraine prevention. However, real-life data are still lacking and overlook the impact of galcanezumab on those different migraine facets strongly contributing to migraine burden. Herein we report the clinical experience from an Italian real-world setting using galcanezumab in patients with migraine experiencing previous unsuccessful preventive treatments.

**Methods:**

Forty-three patients with migraine and failure of at least 3 migraine preventive medication classes received monthly galcanezumab 120 mg s.c. At the first administration and after 3 and 6 months, patients underwent extensive interviews to assess clinical parameters of disease severity. Furthermore, validated questionnaires were administered to explore migraine-related disability, impact, and quality of life as well as symptoms of depression or anxiety, pain catastrophizing, sleep quality and the ictal cutaneous allodynia.

**Results:**

After the third and the sixth administration of monthly galcanezumab 120 mg s.c., headache attacks frequency reduced from 20.56 to 7.44 and 6.37 headache days per month, respectively. Moreover, a significant improvement in headache pain intensity (from 8.95 to 6.84 and 6.21) and duration (from 9.03 to 3.75 and 2.38) as well as in scores assessing migraine related disability and impact, depressive and anxious symptoms, and pain catastrophizing was observed. Furthermore, we demonstrated a significant reduction in the values of “whole pain burden”, a composite score derived from the product of the average of headache frequency, intensity, and duration in the last three months.

**Conclusion:**

Real-world data support monthly galcanezumab 120 mg s.c. as a safe and effective preventive treatment in reducing headache frequency, intensity, and duration as well as comorbid depressive or anxious symptoms, pain catastrophizing and quality of life in both episodic and chronic migraine patients with previous unsuccessful preventive treatments. Furthermore, we demonstrated that monthly galcanezumab 120 mg s.c. is able to induce a significant improvement in the scores of “whole pain burden”. The latter is a reliable and easy-to-handle tool to be employed in clinical setting to evaluate the effectiveness of preventive drugs (in this case, galcanezumab) or when the decision of continuing the treatment with anti-CGRP mAbs is mandatory.

**Supplementary Information:**

The online version contains supplementary material available at 10.1186/s10194-022-01436-6.

## Background

The last years represented a breakthrough in migraine preventive treatments due to the approval of specific monoclonal antibodies targeting the trigeminal sensory calcitonin gene-related peptide (CGRP) or its receptor [1]. Therefore, a new era has been opened in migraine treatment previously dominated by repositioning non-specific drugs often discovered by serendipity and characterized by non-optimal efficacy and tolerability, strongly affecting the therapeutic adherence [2–4]. Among anti-CGRP monoclonal antibodies (CGRP-mAbs), galcanezumab, able to bind and inhibit CGRP molecules, has been approved by international drug agencies in 2019 and established as effective and safe by 4 Phase III randomized controlled trials (RCTs) in episodic migraine and chronic migraine patients [5–8].

Real-life data from a recent observational Italian study confirmed that galcanezumab is highly effective and well-tolerated for the treatment of high-frequency episodic and chronic migraine patients [9]. Nevertheless, previous real-world observations, focusing almost exclusively on migraine frequency, have partially neglect the impact of galcanezumab on other clinical significant characteristics of migraine phenomenon, such as pain severity, attack duration and response to painkillers, closely associated with the patient’s overall pain experience. Furthermore, although multidimensional effects of CGRP-mAbs in migraine patients have been investigated [10], studies exploring the impact of galcanezumab on different migraine facets such as comorbid psychiatric symptoms, pain coping, cutaneous allodynia, and sleep quality are lacking, to date.

We report the clinical experience from an Italian real-world setting using galcanezumab in a cohort of high frequency and chronic migraine patients with previous unsuccessful treatments. Herein, we focused on galcanezumab effectiveness beyond the mere reduction of headache days per month considering changes in the whole pain burden (derived from the average frequency, intensity and duration of the headaches) as well as in the response to painkillers, comorbid psychiatric symptoms, pain coping, cutaneous allodynia, and sleep quality in these patients.

## Methods

### Study design and participants

This was an observational, prospective, non-randomized, open-label study evaluating the efficacy and safety of galcanezumab as preventive treatment in migraine patients with failure of at least 3 previous preventive strategies. Forty-three patients with episodic high frequency (8 pts) and chronic migraine (35 pts) (according to the International Headache Society criteria) [11] were recruited from the population referred to the Headache Center of the Department of Neurology at the University of Campania “Luigi Vanvitelli” between January 2021 and June 2021 and followed up for six months. We included only migraine patients aged between 18 and 75 years and all patients had received and failed (i.e. lack of efficacy or intolerable side effects) at least three oral preventive medications classes (propranolol or metoprolol, topiramate or valproate, flunarizine, amitriptyline) or onabotulinumtoxin-A (only for chronic migraine patients) due to lack of efficacy or intolerable side effects (see supplementary material 1 for further information about patients selection). Lack of efficacy was defined as no meaningful improvement (< 50% of reduction in headache days/month) in the frequency of headaches after at least three months of treatments as recommended by the European Headache Federation guidelines (6 months for onabotulinumtoxin-A) [12]. Tolerability failure was defined as documented discontinuation due to adverse events at any time of the treatment. Patients were allowed to take other preventive oral medication (alone or in combination) if with a stable dose for at least three months but with a stable dose for all the duration of galcanezumab treatment. The headache frequency (defined as the monthly mean of headaches) as well as the headache intensity and response to painkiller were evaluated by reviewing papery standardized headache diaries at baseline (i.e. the three months before starting the treatment with galcanezumab) and during the galcanezumab treatment. *Specifically, all patients fill in a paper headache diary consisting of a table with the days of the month (up to 31 days) on the abscissa and the hours of the day (up to 24 hours) on the frames. The patient should mark the onset and the end of every attacks and the time of pain-killer intake to allow us the calculation of both the attacks duration and the response to the symptomatic drugs, and also in order to evaluate the reduction of painkiller intake/month. In the case of headaches present at both the time of falling asleep and waking up, the night hours were considered as attack hours. Moreover, the patient inserted the intensity of the attacks (according to the 11-point NRS scale) (see supplementary material* *2**for the headache diary used by the patients).* These data were used also to assess a composite score that we defined as “whole pain burden”, derived from the product of headache frequency (i.e. mean of attacks per month in the last three month) per headache intensity (mean of NRS values) per headache duration (mean headache hours when treated). As an example, consider a patient who has - in the last three months - an average frequency of 9 headache days per month whit an average intensity of 8/10 on the NRS scale lasting an average of 6 hours. In this case, the “whole pain burden” score for the last three months would be calculated as product of 9 × 8 × 6 that is 432. All patients received galcanezumab subcutaneous (s.c.) injection, with the first loading dose of 240 mg and then every month with 120 mg. At the first administration (T_0_), at the end of the third (T_1_) and the sixth month (T_2_) of galcanezumab treatment, the headache diaries were analyzed to assess headache days per month, migraine attacks per month, pain intensity (assessed by numerical rating scale [NRS]), headache duration (mean headache hours when treated), response to painkiller (pain-free after two hours), acute pain medication intake. Moreover, all patients underwent an extensive interview aimed to explore a) migraine-related disability (MIDAS) and impact by Headache Impact Test (HIT-6), b) the presence of comorbid depression and anxiety by the Beck Depression Inventory-II (BDI-II), Hamilton Depression Rating Scale (HDRS), and Hamilton Anxiety Rating Scale (HARS); c) quality of sleep by the Medical Outcomes Study (MOS) Sleep Scale d) quality of life by the migraine-specific quality-of-life questionnaire (MSQ), e) Allodynia Symptom Checklist-12 (ASC-12) and f) Pain Catastrophizing Scale (PCS) [13–21]. During the six months period of observation, all adverse events (AE) related to the drug were recorded and used as a safety measure.

The protocol was reviewed and approved by the Ethical Committee of the University of Campania “Luigi Vanvitelli”. Each patient gave a free, informed consent for participation in the study and the analysis and publication of the protocol data (code 30564/20). The study was done according to the Strengthening the Reporting of Observational Studies in Epidemiology (STROBE) guidelines [22].

### Primary outcomes

The primary endpoints of the study were the reduction in monthly headache days, pain intensity, attacks duration as well as in the “whole pain burden” score, at the end of the third (T^1^) and of the sixth month (T^2^) of galcanezumab treatment compared with the baseline.

### Secondary outcomes

Secondary endpoints, considering the baseline compared with the third and the sixth monthly galcanezumab administrations, were i) proportion of patients who achieved at least 50% and 75% reduction from their individual baseline in monthly headache days, ii) proportion of patients who achieved at least 50% reduction from their individual “whole pain burden” score, iii) change in monthly painkillers intake as both days with painkillers intake and number of painkillers intake (i.e. as pills/tablets/injection) iv) change in MIDAS, HIT-6, MSQ, BDI-II, HDRS, HARS, PCS, MOS sleep scale, and ASC-12 scores, and, finally, iv) the percentage of patients converting from chronic migraine to episodic migraine as well as v) the percentage of patients converting from not-responders to responders to pain killers.

### Statistical analysis

All demographic and clinical data were checked for normality using Shapiro–Wilk test. Continuous variables are reported as mean ± standard deviation (SD), categorical variables are expressed as median ± interquartile range, rates values are reported as subjects-counts and percentage. In all subjects, the paired *t*-test was used to compare the mean headache days per month, mean pain intensity, mean headache attack duration and “whole pain burden” score at baseline (T^0^) and at the end of the third (T^1^) and the sixth month (T^2^) of galcanezumab treatment. Wilcoxon rank test was used to compare HIT-6, MIDAS, MSQ, BDI-II, HDRS, HARS, PCS, MOS sleep scale, and ASC-12 scores. Statistical significance was set at *p* < 0.003 after Bonferroni correction for multiple comparison (0.05/14 comparisons). Due to the observational approach of the study, the power analysis has not been performed. A squared partial correlations analysis has been conducted to explore whether included parameters (headache attacks frequency, pain intensity and attack duration) explain the variance of “whole pain burden” score. To explore the validity of the construct of the “whole pain burden” composite score, the Spearman’s rank correlation was used to determine the correlation of the MSQ to “whole pain burden” composite score at baseline. Multivariate regression analysis was conducted, including clinical parameters of disease severity (disease duration, baseline headache days per month, pain intensity, attack duration, MIDAS, HIT-6, MSQ, HARS, HDRS, PCS scores, ASC-12) to determine the independent predictors of response to galcanezumab treatment (statistical significance was set at *p* < 0.05). All analyses were performed using Stata version-16 (StataCorp, College Station, TX, USA).

## Results

### Demographic and baseline headache characteristics

The whole population consisted of 43 migraine patients with a history of treatment failures to at least three preventive medication classes (3.7 ± 0.85, see supplementary material 3 for further information), considered as lack of efficacy or intolerable side effects leading to treatment discontinuation. The majority of patients were female (88.37%), with a mean age of 46 ± 13 years. The average time since migraine onset was 25.76 (± 15.35) years. The average frequency of attacks/month at baseline was 18.33 (± 7.15), while the average headache days/month was 20.56 (± 7.55). In our population, there were 35 (81.40%) chronic migraine patients, while 8 (28.60%) were high frequency episodic migraine patients. Furthermore, among chronic migraine patients, 32 (91.43%) patients presented medication overuse headache (MOH). Demographic and baseline headache characteristics of patients included in the study are reported in Table 1.

**Table 1:** Baseline demographic and clinical parameters

Characteristics	*N* = 43
Age, mean ± SD	46 ± 13
Gender, n (%)	
Male	5 (11.63%)
Female	38 (88.37%)
Age at migraine onset (years), mean ± SD	18.83 ± 10.38
Disease duration (years) mean ± SD	25.76 ± 15.35
Concurrent oral preventive treatments, n (%)	12 (27.91%)
Monotherapy	7 (16.28%)
Polytherapy	5 (11.63%)
Headache days/month, mean ± SD	20.56 ± 7.55
Migraine attacks/month, mean ± SD	18.33 ± 7.15
Chronic migraine pts. n (%)	35 (81.40%)
Preventive classes failure, mean ± SD	3.7 ± 0.85
Pain intensity (NRS), mean ± SD	8.95 ± 1.00
Attack duration (hours), mean ± SD	9.03 ± 11.09
Whole pain burden, median ± IQR	400 ± 1953
Pain free 2 hours after painkiller intake, n (%)	25 (58.14%)
Days with painkiller intake, mean ± SD	18.40 ± 8.37
Painkiller intake/month, mean ± SD	22.58 ± 12.33
MOH, n (%)	32 (91.43% of chronic migraine patients)
HIT-6, median ± IQR	69 ± 9
MIDAS, median ± IQR	90 ± 65
MSQ, median ± IQR	75.71 ± 28.57
BDI-II, median ± IQR	13.5 ± 15
HDRS, median ± IQR	15 ± 10
HARS, median ± IQR	13 ± 9
Sleep problem INDEX II, median ± IQR	30.56 ± 22.22
ASC-12, median ± IQR	6 ± 7
PCS, median ± IQR	36.5 ± 14

Values are mean ± standard deviation (SD) or ± interquartile range (IQR) or number (%)

*n* number, *pts.* patients, *NRS* numerical rating scale, *HIT-6* headache impact test-6, *MIDAS* migraine disability assessment scale, *MOH* medication overuse headache, *MSQ* migraine-specific quality-of-life questionnaire, *BDI II* Beck Depression Inventory, *HDRS* Hamilton Depression Rating Scale, *HARS* Hamilton Anxiety Rating Scale, *ASC-12* Allodynia Symptom Checklist-12, *PCS* Pain Catastrophizing Scale

### Primary endpoints

Headache attack frequency reduced from 20.56 to 7.44 and 6.37 headache days per month after the third and the sixth administration of monthly galcanezumab 120 mg s.c., respectively (*p* < 0.001).

Headache attack pain intensity reduced from a NRS score of 8.95 to 6.84 and 6.21 after the third and the sixth administration of monthly galcanezumab 120 mg s.c., respectively (*p* < 0.001).

Headache attack duration (treated) reduced from a 9.03 to 3.75 and 2.38 headache hours after the third and the sixth administration of monthly galcanezumab 120 mg s.c., respectively (*p* < 0.001).

**Fig. 1:**
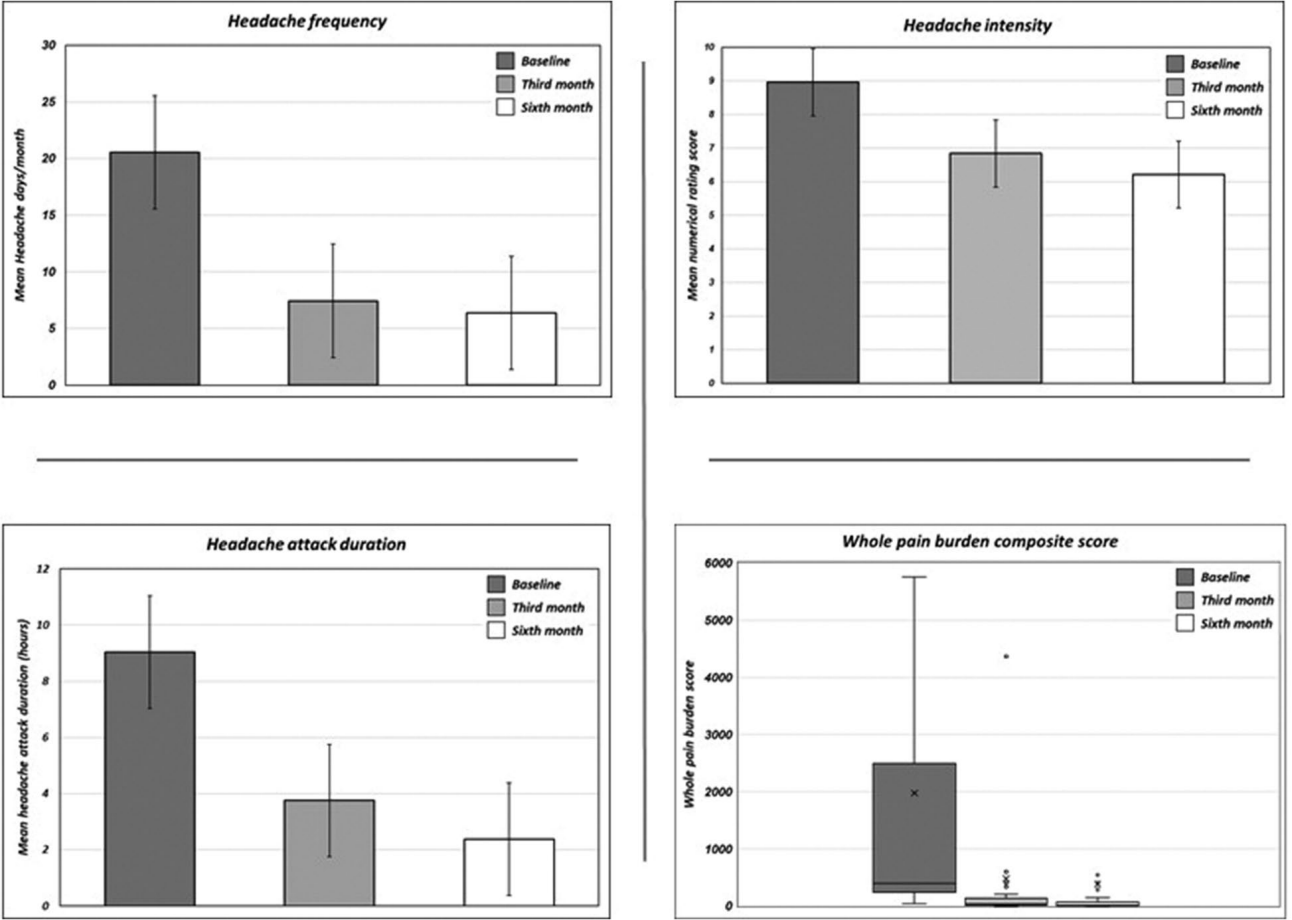
Primary outcome: headache attacks frequency, intensity, duration and “whole pain burden” score at baseline (T^0^), and at the end of the third (T^1^) and sixth month (T^2^) of galcanezumab administrations

The “whole total pain burden” score decreased from baseline 1975.05 to 477.05 and 383.72 after the third and the sixth administration of monthly galcanezumab 120 mg s.c., respectively (*p* < 0.001) (See Fig. 1 and Table 2 further information).

**Table 2:** Efficacy endpoints after the third and sixth monthly galcanezumab administrations (*n* = 43)

Outcomes	Baseline	Administration	
		Third	Sixth
Headache days per month (mean ± SD)	20.56 ± 7.55	7.44 ± 7.47* (*p* < 0.001)	6.37 ± 7.30* (*p* < 0.001)
≥50% Reduction in headache days/month from baseline n (%)		31 (72.09)	32 (74.42)
≥75% Reduction in headache days/month from baseline n (%)		19 (44.19)	24 (55.81)
Conversion from chronic (35 pts) to episodic migraine n (%)		26 (74.29)	28 (80.00)
Low- frequency n (%)		23 (88.46)	25 (89.29)
High- frequency n (%)		3 (11.54)	3 (10.71)
Conversion from high (8 pts) to low-frequency episodic migraine n (%)		7 (87.5)	8 (100)
Pain intensity (NRS) (mean ± SD)	8.95 ± 1.00	6.84 ± 1.69* (*p* < 0.001)	6.21 ± 2.07* (*p* < 0.001)
Attack duration (hours) (mean ± SD)	9.03 ± 11.09	3.75 ± 8.59* (*p* < 0.001)	2.38 ± 4.97* (*p* < 0.001)
“Whole pain burden” score (median ± IQR)	400 ± 1953	35 ± 115.5* (*p* < 0.001)	17.5 ± 56* (*p* < 0.001)
Pain free 2 hours after painkiller intake n (%)	25 (58.14)	30 (69.77)	33 (76.74)
Days with painkiller intake (mean ± SD)	18.40 ± 8.37	6.74 ± 7.45* (*p* < 0.001)	6.12 ± 7.23* (*p* < 0.001)
Painkiller intake/month (mean ± SD)	22.58 ± 12.33	8.65 ± 11.11* (*p* < 0.001)	7.05 ± 8.16* (*p* < 0.001)
Pts converting from not-responders [18] to responders n (%)		5 (27.78)	8 (44.44)
n(%) of responders to painkiller (pain free at two hours)	24 (55.81)	30 (69.77)	31 (72.09)
MIDAS (median ± IQR)	90 ± 65	20 ± 27* (*p* < 0.001)	15.5 ± 30.5* (*p* < 0.001)
HIT-6 (median ± IQR)	69 ± 9	58 ± 8* (*p* < 0.001)	57.5 ± 9.5* (*p* < 0.001)
MSQ (median ± IQR)	75.71 ± 28.57	30 ± 40* (*p* < 0.001)	28.57 ± 26.43* (*p* < 0.001)
BDI-II (median ± IQR)	13.5 ± 15	8 ± 10 (*p* = 0.019)	8 ± 10.5* (*p* = 0.003)
HDRS (median ± IQR)	15 ± 10	10 ± 7.25 (*p* = 0.014)	10 ± 7* (*p* = 0.002)
HARS (median ± IQR)	13 ± 9	11.5 ± 7.5* (*p* = 0.002)	10 ± 9* (*p* < 0.001)
PCS (median ± IQR)	36.5 ± 14	25 ± 14.5* (*p* = 0.002)	21 ± 13* (*p* < 0.001)
Sleep problem INDEX-II (median ± IQR)	30.56 ± 22.22	27.78 ± 9.73 (*p* = 0.443)	27.78 ± 18.03 (*p* = 0.183)
ASC-12 (median ± IQR)	6 ± 7	4 ± 8.5 (*p* = 0.169)	4 ± 8 (*p* = 0.01)

Values are mean ± standard deviation (SD) or ± interquartile range (IQR) or number (%)

*statistically significant (in comparison with baseline)

*n* number, *NRS* numerical rating scale, *pts.* patients, *MIDAS* migraine disability assessment scale, *HIT-6* headache impact test-6, *MSQ* migraine-specific quality-of-life questionnaire, *BDI II* Beck Depression Inventory, *HDRS* Hamilton Depression Rating Scale, *HARS* Hamilton Anxiety Rating Scale, *PCS* Pain Catastrophizing Scale, *ASC-12* Allodynia Symptom Checklist-12

### Secondary endpoints

**Fig. 2:**
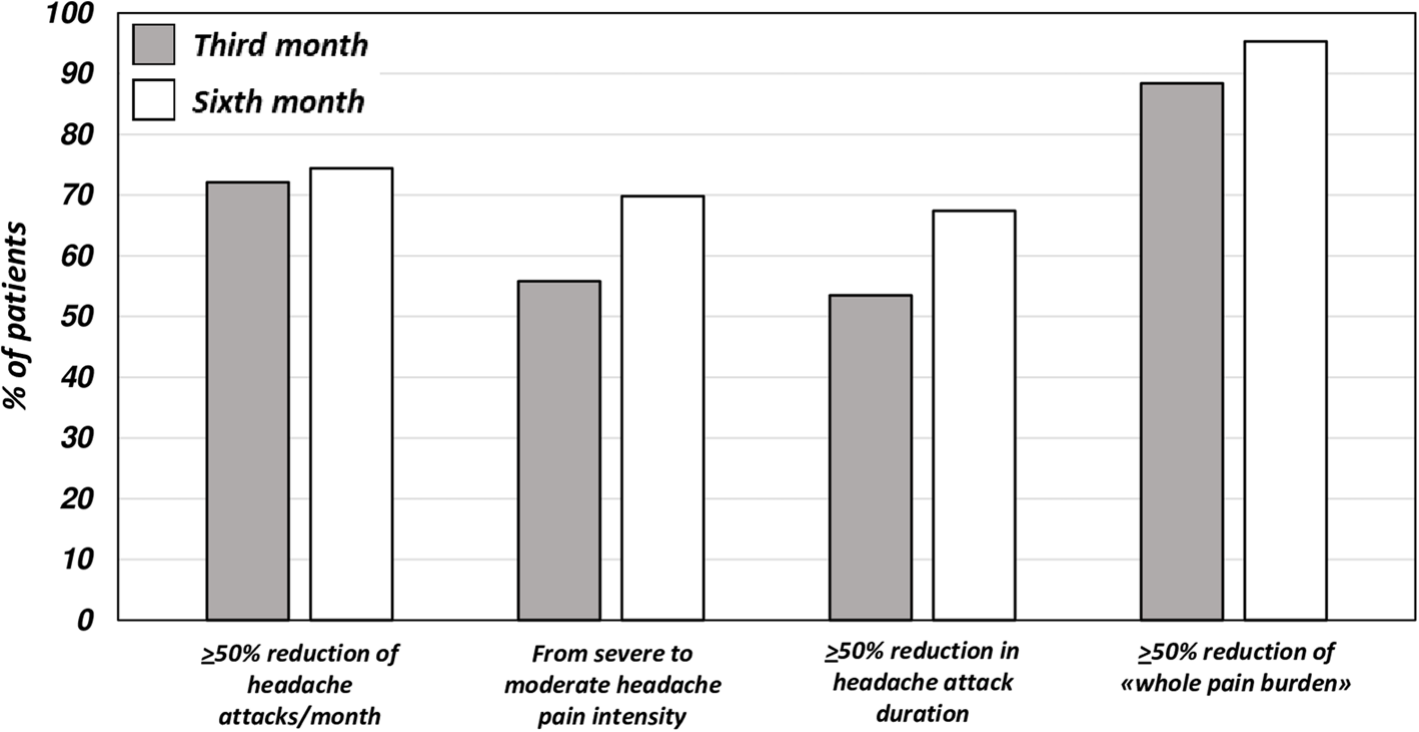
Percentage of patients reporting a > 50% reduction in headache attacks frequency, pain intensity, headache attacks duration and “whole pain burden” score at the end of the third (T^1^) and sixth month (T^2^) galcanezumab administrations

A reduction of at least 50% of headache days per month was reported by 72.09% and the 74.42% of migraine patients after the third and the sixth administration of monthly galcanezumab 120 mg s.c., respectively. A reduction of at least 75% was reported by the 44.19% and 55.81% of migraine patients after the third and the sixth administration respectively. A reduction of at least 50% of the “whole total pain burden” score was reported by 88.37% and 95.35% of migraine patients after the third and the sixth administration of monthly galcanezumab 120 mg s.c., respectively. Particularly, a reduction of at least 75% was reported by 76.74% and 88.37% of migraine patients after the third and the sixth administration respectively. (See Fig. 2 and Table 2 for further information).

A significant reduction was observed in the number of monthly days with painkillers intake from baseline 18.40 to 6.74 and 6.12 as well as in total painkillers intake (i.e. as number of pills/tablets/injection per month) from baseline 22.58 to 8.65 and 7.05 after the third and the sixth administration of monthly galcanezumab 120 mg s.c. respectively (*p* < 0.001), moreover, we observed a reduction in the number of patients with MOH (from 32 to 8 and 8 after the third and the sixth administration).

**Fig. 3:**
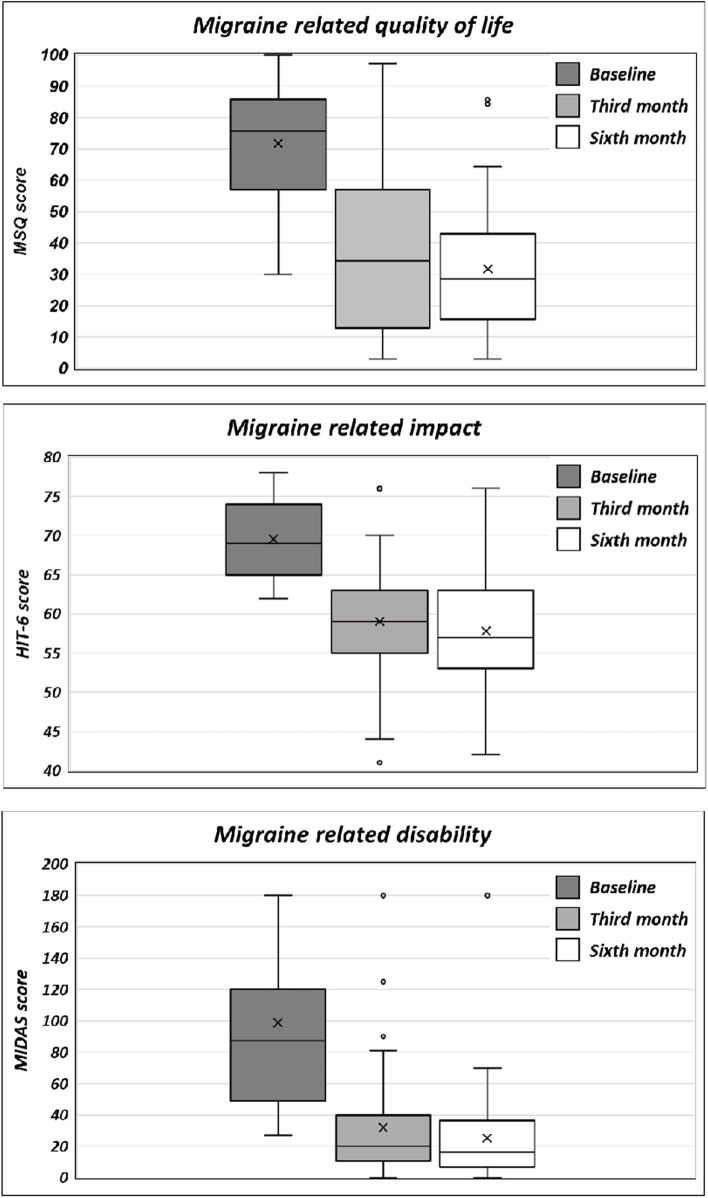
Migraine-related disability, impact on daily living and quality of life scores at baseline (T^0^), and at the end of the third (T^1^) and of the sixth month (T^2^) of galcanezumab administration

Significant improvements were observed in MIDAS and HIT-6 (*p* < 0.001) and impact on daily living assessed by MSQ (*p* < 0.001) after the third and sixth administration when compared with baseline (see Fig. 3 and Table 2 for further information).

**Fig. 4:**
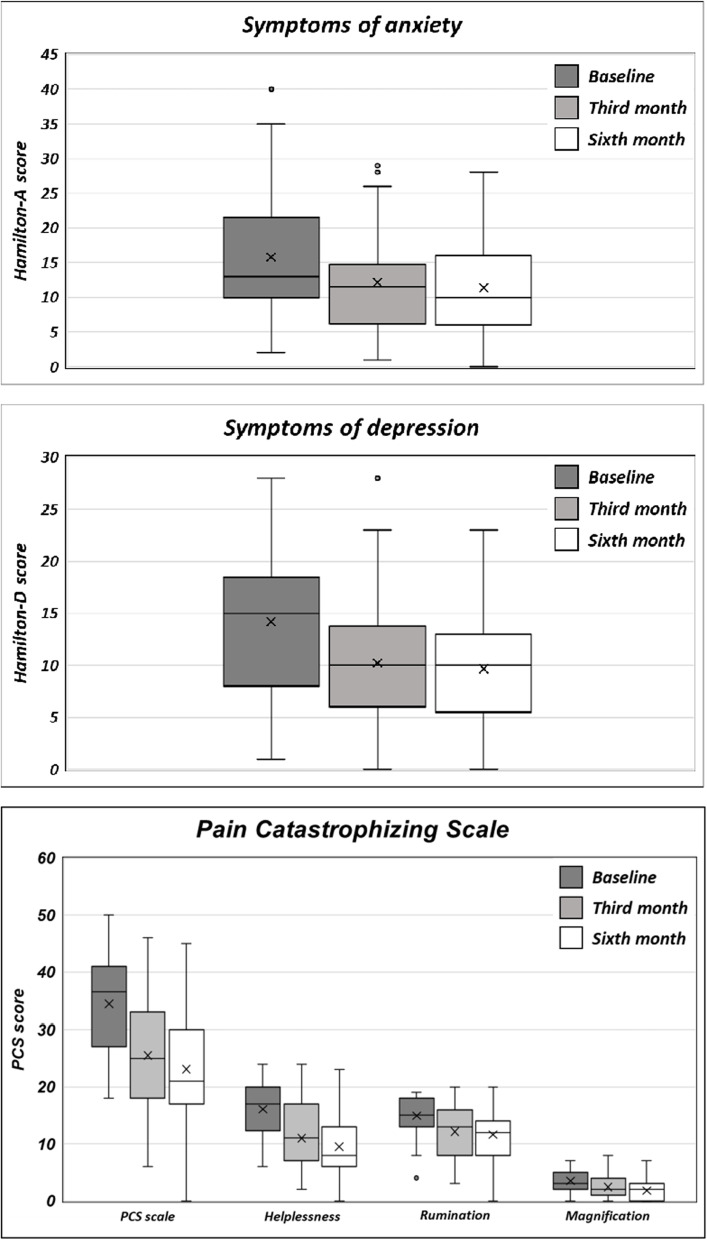
Symptoms of depression, anxiety and pain catastrophizing scores at baseline (T^0^), and at the end of the third (T^1^) and sixth month (T^2^) of galcanezumab administrations

Anxiety symptoms assessed by HARS as well as PCS scores (in the three sub-domains of rumination, magnification, and helplessness) significantly improved from baseline since the third administration (*p* < 0.001) (see Fig. 4 and Table 2 for further information).

No change was observed in quality of sleep assessed by the MOS sleep scale from baseline after the third and sixth administration.

Cutaneous allodynia symptoms experienced during the attacks (assessed by ASC-12) improved after the third and sixth administration but did not reach statistical significance after correction for multiple comparison (*p* = 0.01).

**Fig. 5:**
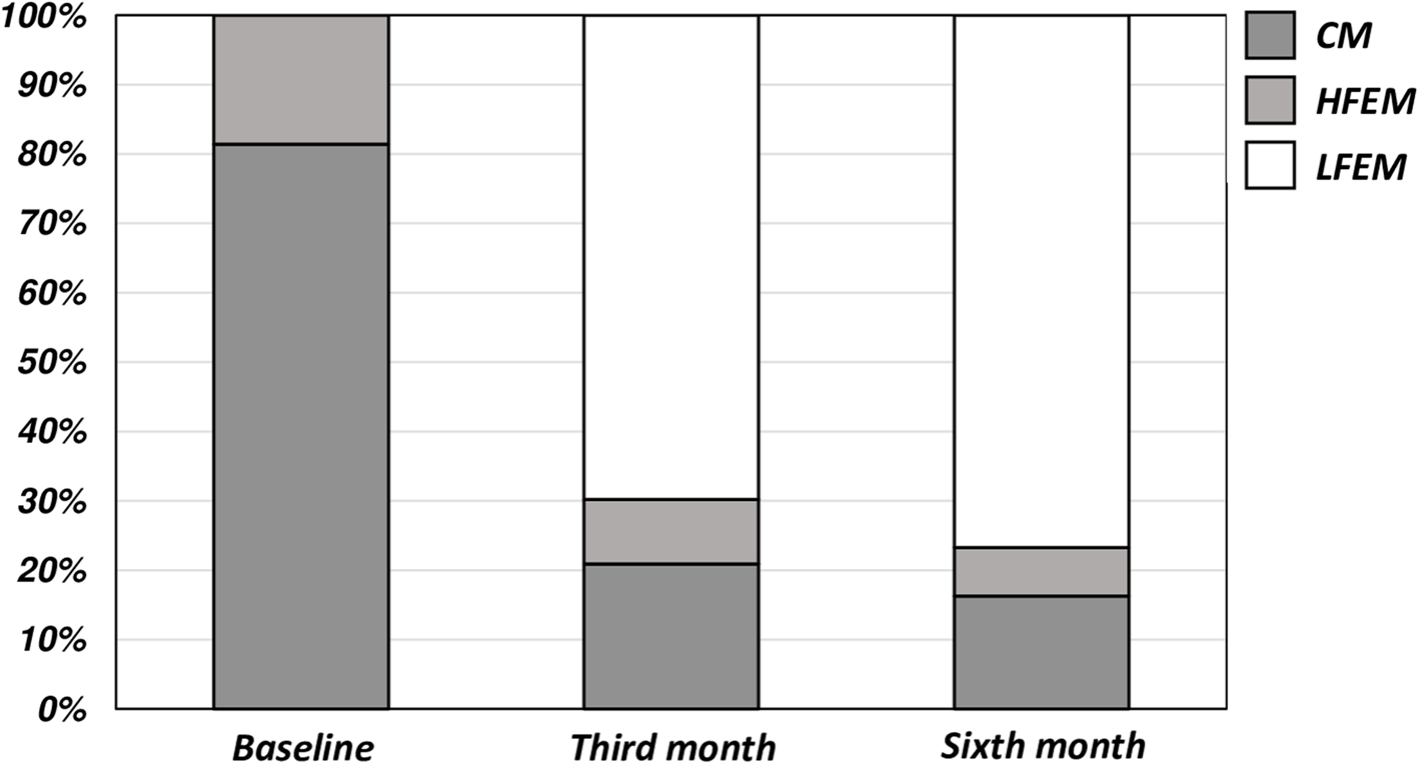
Percentage of patients with chronic migraine, high frequency episodic migraine and low frequency episodic migraine at baseline (T^0^), and at the end of the third (T^1^) and of the sixth month (T^2^) of galcanezumab administrations

The 74.29% and 80% (*n* = 26 and 28) of chronic migraine patients converted to episodic migraine (11.54% and 10.71% high frequency and 88.46% and 89.29 low frequency), while the 87.5% and 100% (*n* = 7 and 8) of high frequency migraine patients converted to low frequency migraine after the third and sixth administration (see Fig. 5 for further information).

The percentage of migraine patients responders to painkillers (i.e. pain free within 2 hours) increased from the 55.81% to 69.77% and 72.09% after the third and the sixth administration of monthly galcanezumab 120 mg s.c., respectively.

Analysis of *b* coefficients of the multivariate regression analysis found baseline high headache intensity and PCS scores as statistically significant prognostic factors of response to galcanezumab treatment (coefficient: − 3.44 *p* = 0.026; coefficient: 0.39, *p* = 0.048) (i.e., the higher headache pain intensity at baseline, the lower the number of headache days after 6 month galcanezumab treatment; the higher the PCS scores at baseline, the higher the number of headache attacks per month after 6 month galcanezumab treatment) (See Table 3 for further information).

**Table 3:** Multiple regression analyses assessing which baseline clinical parameters is associated with a better clinical response to monthly galcanezumab 120 mg administration after 6 months of treatment (dependent variable: headache days/month after 6 months)

Independent variable	Coefficient b	*p*-value	SE	95% CIs for b Coefficient	
				Lower	Upper
*Multiple regression*					
Disease history (years)	−0.17	0.604	0.08	−0.34	− 0.00
Previous treatments failures	1.85	0.288	1.69	−1.71	5.42
Headache days/month	0.09	0.779	0.32	−0.59	0.77
Headache attacks intensity (NRS)	−3.44	**0.026**	1.41	−6.42	−0.46
Headache attacks duration (hours)	0.1	0.780	0.35	−0.64	0.83
Whole pain burden	−0.00	0.887	0.00	−0.00	0.00
MIDAS	0.04	0.168	0.03	−0.02	0.10
HIT-6	−0.23	0.443	0.29	−0.84	0.39
MSQ	−0.7	0.375	0.08	−0.23	0.09
PCS	0.39	**0.048**	0.22	−0.08	0.86
ASC-12	0.29	0.368	0.31	−0.37	0.94
BDI-II	−0.12	0.670	0.28	−0.71	0.47
HDRS	−0.3	0.944	0.35	−0.77	0.72
HARS	0.00	0.987	0.28	−0.58	0.59

Note. *SE* Standard Error; a, Model F = 1.97, *p*-value 0.048, R^2^ = 0.62 (Nagelkerke)

*NRS* numerical rating scale, *pts.* patients, *MIDAS* migraine disability assessment scale, *HIT-6* headache impact test-6, *MSQ* migraine-specific quality-of-life questionnaire, *BDI II* Beck Depression Inventory, *HDRS* Hamilton Depression Rating Scale, *HARS* Hamilton Anxiety Rating Scale, *PCS* Pain Catastrophizing Scale, *ASC-12* Allodynia Symptom Checklist-12

### “Whole pain burden” composite score: partial correlation analysis and convergent validity

The squared partial correlation analysis showed that each “whole pain burden” parameter among headache attacks frequency (*p* < 0.001), pain intensity (*p* = 0.04) and attack duration (*p* < 0.001) explain the variance of “whole pain burden” score. The “whole pain burden” composite score exhibited a significant correlation with MSQ score at baseline (r = 0.62; *p* < 0.001).

### Safety and tolerability

Treatment-related AE were consistent with the well-known tolerability profile of galcanezumab. Out of the pain in the site of injection, 10 pts. (23.26%) reported in the course of treatment systemic AE. Among these, 7 pts. (16.27%) reported constipation, 3 pts. (6.98%) reported fatigue, and 1 pts. (2.32%) reported acrocyanosis. None of the above-mentioned AE led to discontinue the treatment. No serious AE were observed (by physical examination) or reported by patients.

## Discussion

In the present study, we report the effectiveness of monthly galcanezumab 120 mg s.c. administration in a cohort of high frequency and chronic migraine patients, with at least 3 previous preventive treatments failure, during a six-month period. Beyond the improvement of headache frequency, intensity and duration, we observed a significant improvement in scores assessing migraine related disability and impact, depressive and anxious symptoms, pain coping, cutaneous allodynia and quality of life. Furthermore, we considered a composite score derived from the product of headache frequency, headache intensity, and headache duration (the last as a proxy of response to painkillers) that, for sake of shortness, we named “whole pain burden”.

Migraine is recognized as the second-highest cause of years lived with disability, the first in patients aged between 15 and 49 years [23] and undertreatment strongly affect migraine related disability, especially in patients with high frequency or chronic migraine [24–26]. Therapeutic approach to these patients involves the use of preventive strategies based, until few years ago, only on “repositioning drugs” characterized by unsatisfactory responses but, above all, by non-excellent tolerability profiles strongly affecting patients’ adherence to treatment [3, 27, 28].

The availability of CGRP-mAbs as novel specific, effective and well-tolerated therapeutic option has represented a breakthrough in migraine preventive treatment [29, 30]. Among CGRP-mAbs, galcanezumab efficacy and safety in migraine prevention has been provided by evidences from rigorously controlled, randomized, double-blind, placebo-controlled, phase 2 or 3b studies in patients with episodic and chronic migraine as well as in both patients with previous migraine preventive medications failure (the latter data confirmed also in an Italian real-life experience) [5–9].

Nevertheless, previous galcanezumab studies did not deeply investigate the impact of this drug on overlooked aspects that strongly contribute to migraine burden, such as coexistent depressive and anxiety symptoms, the cognitive strategies to face with pain, experience of cutaneous allodynia as well as sleep quality.

Herein, we demonstrated the effectiveness of galcanezumab over a six-month period in a group of 43 migraine patients with documented failure to at least three migraine preventive medication classes. Specifically, we observed a decreased number of headache days per month from 20.56 to 7.44 and 6.37 with a reduction of at least 50% of headache days per month in 72.09% and 74.42% of patients after the third and the sixth administrations of monthly galcanezumab, respectively. These data are in line with those emerging from a recent prospective study showing that anti-CGRP mAbs induce a significant reduction in monthly migraine days from the first month of treatment, followed by a further slight decrease until month 6. This may suggest that at least 6 months of galcanezumab treatment are needed to reach the therapeutic plateau necessary to counteract the neurobiological mechanisms underpinning CGRP desensitization [31].

Beyond mere pain related parameters, several patient-reported outcomes (PROs) have been considered. In this context, the high headache impact (by means of HIT-6) and the severe disability (by means of MIDAS) as well as the critical impairment in migraine-specific quality of life (by means of MSQ) registered at the baseline showed a significant improvement after the third and, even more, the sixth monthly galcanezumab administration in these patients.

Furthermore, at baseline 46.51% and 27.91% of patients showed mild depression (by mean of HDRS and the self-administrated BDI-II) and moderate anxiety comorbidities (by mean of HARS) respectively. Statistically significant improvements have been observed since the third administration of monthly galcanenumab leading to mean scores values consistent with the absence of both depressive and anxiety contents. It is noteworthy that depressive and anxious symptoms are able to worsen migraine attacks increasing the rates of progression to chronic migraine and making migraine treatment more challenging, reducing quality of life and increasing the overall disease burden [32–34]. Since the greater is the burden of migraine the higher is the probability of experiencing depressive or anxious symptoms [35], we argue that galcanezumab related improvement of migraine frequency, severity and responsiveness to pain-killer as well as headache impact, disability and quality of life may reflect on the comorbid psychiatric symptoms.

Beyond depressive and anxiety symptoms, the 58.14% of our patients showed PCS scores at baseline above the cut-off value, witnessing negative orientation toward actual or anticipated pain experience. A significant reduction in PCS scores, in all sub-domains, was found since the third monthly galcanezumab administration.

Maladaptive pain coping strategies, consisting on negative cognitive and affective behavior in response to pain, such as the so-called “pain catastrophizing”, are well-reported in migraine patients [36]. In particular, rumination of pain related thoughts, magnification of pain experience, and helplessness about it are strong predictors of headache outcomes and significantly associated with disability. Moreover, “pain catastrophizing” is associated with migraine chronicization, poorer treatment response, increased medical consultation, impaired functioning and psychological distress leading to reduced health-related quality of life [37–39].

Furthermore, in our patients group, we assessed sleep quality using the MOS-sleep scale, a self-administered scale able to evaluate 6 different disturbances (i.e. difficulty falling asleep and maintaining sleep, daytime sleepiness, respiratory disorders, presence of ronchopathy, amount of sleep). The incidence and prevalence of sleep disorders are significantly higher in migraine patients when compared to general population and sleep-wake rhythm and the quality of sleep abnormalities surely represent trigger factors for migraine attacks [40, 41]. In particular, insomnia, the most common sleep complaint among migraine patients, has been observed in 40% of episodic migraine patients and in almost 70% of chronic migraine patients, half of which also reporting snoring during sleep [42]. Based on the present observations, no changes were found in sleep patterns considering the comprehensive “sleep problem index” in the course of galcanezumab treatment. We can speculate on the possibility that galcanezumab, similarly to other anti-CGRP monoclonal antibodies [10], may be able to improve sleep-wake rhythm abnormalities although longer periods are needed.

In our patient sample, 31% reported ictal cutaneous allodynia (CA) at baseline. After the third and sixth monthly galcanezumab administrations we noted a remarkable reduction of ASC-12 scores (*p* = 0.01) although it became not statistically significant after correction for multiple comparison. Nevertheless, we can argue that galcanezumab-induced peripheral CGRP inhibition may be able to indirectly inhibit the central trigemino-thalamic pathway sensitization known to be involved in ictal CA and, in turns, putatively inhibiting chronification mechanisms. It is well-known that CA is reported in about two-thirds of migraine patients as the perception of pain induced by trivial stimuli to normal skin, during or between headache episodes [43]. CA is known to represent a negative predictor of response to both acute and preventive medications and, overall, a risk factor for migraine chronification [44]. Future studies on a larger cohort of patients or aimed to assess CA changes after longer periods of galcanezumab treatment may strongly substantiate our present observations.

Finally, in line with previous observation, a significant reduction was observed in the number of monthly days with painkillers intake as well as in total painkillers intake with a significant reduction in the number of chronic migraine patients with MOH in the course of galcanezumab administration [45].

One of the main findings of the present study is the identification of a composite score derived from the product of headache frequency per headache intensity per headache duration (the last as a proxy of response to painkillers) in the last three months, that for sake of shortness, we named “whole pain burden” score. Previously, a composite measure has been already employed to fully assess the potential benefit of migraine treatment strategies (the so-called “total pain burden”) obtained by multiplying duration (hours) of migraine headache and maximum pain severity (0 = none, 1 = mild, 2 = moderate, 3 = severe) for each migraine headache day and summing these over the days in a month [46]. The “total pain burden” score was created to better reflect the individual’s migraine experience and aimed to patient-centric discussions regarding treatment expectations when clinicians are evaluating options for migraine prevention. However, calculating “total pain burden” scores in clinical practice is really complex and time consuming since it is based on day-by-day calculation of headache hours, severity and duration. In other terms, we believe that the “total pain burden” **score** can be a useful tool in RCT settings but seems to be inadequate to the times of the real-world setting. Furthermore, the use of a 4-point scale to assess migraine attack severity to calculate the “total pain burden” is affected by a reduced sensitivity in detecting pain intensity changes. Therefore, aware of the relevance of a unique score in clinical practice, more adherent to the personal headache experience as well as to the burden of migraine on the patients’ life, we decided to consider a “whole pain burden” composite score derived from the combination of the average values of headache frequency, intensity (using a 11 point NRS), and duration. This approach let us to observe, after the third and sixth galcanezumab administration, a significant reduction of the “whole pain burden” score in migraine patients.

Interestingly, “whole pain burden” score reduction > 50% has been found in a significant percentage of patients (73.68% and 76.93% respectively after the third and the sixth monthly galcanezumab 120 mg sc administrations) with < 50% reduction in headache days per month and, therefore, to be considered, strictly speaking, poor responders to galcanezumab treatment. The partial correlation analysis demonstrated that each element employed to calculate the “whole pain burden” score significantly contributes to its determination. The value of the “whole pain burden” composite score, as a comprehensive tool to evaluate the complexity of headache experience as well as the potential benefit of migraine preventive treatments on the quality of life, is further supported by a convergent validity witnessing the correlation between the “whole pain burden” scores and a parameter of quality of life related to migraine as the MSQ.

It could be argued that a limitation of the “whole pain burden” composite score is the low weight attributed to the number of symptomatic drugs administered. On the other hand, we would underline that the “whole pain burden” is a composite score aimed to an effective and rapid evaluation of the treatment-related changes of the experience of headache. It is noteworthy that some important aspects beyond the pain experience itself are overlooked by the “whole pain burden” composite score, such as the number of pain-killers intake and the impact of the migraine accompanying symptoms. Although aware of the limits related to the patients reported outcomes (i.e. intra-personal variation in reliability, persisting in repeated applications, decrease motivation to respond etc.) [47], we cannot exclude that in the future, a more complex scores, encompassing the overall migraine experience, could be proposed. However, herein we have observed a notable reduction in both the number of mean monthly days with painkillers intake and total painkillers intake after the third and sixth galcanezumab administration compared with baseline.

In the present real-life experience, there were no patients reporting serious AE or willing to discontinue treatment due to poor tolerability, although a low percentage of migraine patients experienced, beside the pain in the site of injection, constipation (16.27%), acrocyanosis (6.98%), nausea (2.32%), supporting galcanezumab as highly effective preventive treatment with a very low percentage of side-effects.

Linear regression analysis showed that both low pain intensity at baseline and high PCS scores at baseline represent negative predictors of response to galcanezumab treatment. It can be speculated that, as previously demonstrated with Onabotulinumtoxin-A [48], the probability of being a responder to CGRP-targeting drugs may be higher in patients with increased peripheral trigeminal sensitization and CGRP production both leading to more intense headache attacks. Moreover, “catastrophizing” habits and abnormal cognitive and emotional approaches to the pain experience, are already known to be associated with poor response to preventive migraine treatments including CGRP-mAbs [37, 49].

The present study is not free from some limitations. First of all, being a non-randomized open-label study, there was no placebo or active comparator arm [50]. However, open-label studies not necessarily overestimate the effectiveness of treatments especially when effectiveness and safety profiles are well-established, as it is with galcanezumab, in both episodic and chronic migraine. However, registering a persistency of 100% of patients in the absence of changes in preventive medications, we are exempt from unintentional bias related to long-term follow-up.

## Conclusion

Our real-world data support monthly galcanezumab 120 mg s.c. as an effective preventive treatment able to reduce headache frequency, intensity and duration in a significant percentage of migraine patients experiencing previous unsuccessful preventive treatments. In addition, galcanezumab showed a significant effect on disability and impact on daily living related to migraine, as well as on both depressive and anxiety symptoms, health-related quality of life, pain catastrophizing and CA.

Furthermore, we considered a composite measure, the so-called “whole pain burden”, that may be more aligned to the personal experience of pain in patients with migraine. Indeed, the “whole pain burden” may better reflect what clinicians and patients discuss regarding the individual’s pain experience in the course of the attacks. Moreover, our experience suggests that “whole pain burden” is an easy-to-handle tool able to evaluate the effectiveness of preventive drugs for migraine (in this case, galcanezumab) and suitable when the decision of continuing the treatment with anti-CGRP mAbs is mandatory.

## Supplementary Information

**Additional file 1: Supplementary material 1.** Flowchart of patients selection

**Additional file 2: Supplementary material 2.** Headache diary

**Additional file 3: Supplementary material 3.** Table about drug class failure

## Data Availability

The data sets analyzed during the current study are available from the corresponding author on reasonable request.

## Abbreviations

AE: Adverse event
ASC: Allodynia Symptom Checklist
BDI-II: Beck Depression Inventory-II
CA: Cutaneous allodynia
CGRP: Calcitonin gene-related peptide
HARS: Hamilton Anxiety Rating Scale
HDRS: Hamilton Depression Rating Scale
HIT: Headache impact test
mAb: Monoclonal antibody
MIDAS: Migraine-related disability
MOS: Medical Outcomes Study
MSQ: Migraine-specific quality-of-life questionnaire
NRS: Numerical rating scale
PCS: Pain Catastrophizing Scale
PROs: Patients-reported outcomes
RCT: Randomized controlled trials
s.c.: Subcutaneous
SD: Standar deviation
STROBE: Strengthening the Reporting of Observational Studies in Epidemiology
