# Supplementary material for: Galcanezumab effect on “whole pain burden” and multidimensional outcomes in migraine patients with previous unsuccessful treatments: a real-world experience
Source: J Headache Pain. 2022 Jun 13;23(1):69. doi: 10.1186/s10194-022-01436-6 (PMC9195341; doi:10.1186/s10194-022-01436-6)
Supplement: Supplementary file 3 — Additional file 3: Supplementary material 3. Table about drug class failure [file 10194_2022_1436_MOESM3_ESM.docx]

|  | β-Blockers | Tricyclic antidepressants | Anticonvulsivant drugs | Calcium channel blockers | Onabotulinumtoxin-A | Others |
| --- | --- | --- | --- | --- | --- | --- |
| Total migraine pts | 34 | 36 | 38 | 25 | 15 | 10 pts |
| Pts with chronic migraine | 28 | 30 | 31 | 22 | 15 | 7 pts |
| Pts with pisodic migraine | 6 | 6 | 7 | 3 | 0 | 3 pts |

**Supplementary material_3:** Drug class failure (*N* of pts)
